# Supplementary material for: Recovering Ancient Grapevine Cultivars in the Balearic Islands: Sanitary Status Evaluation and Virus Elimination
Source: Plants (Basel). 2022 Jun 30;11(13):1754. doi: 10.3390/plants11131754 (PMC9268875; doi:10.3390/plants11131754)
Supplement: Supplementary file 1 [file plants-11-01754-s001.zip › plants-1773877-supplementary.pdf]

plants MDPI

Supplementary Table S1: Incidence of virus in each cultivar (%). To simplify the interpretation of the results, GLRaV-1,-3 were grouped as single pathogen (GLRaVs), being both viruses associated to the same disease (Grapevine leafroll).

		Virus Incidence		
**Local Cultivars**	**Virus Free**	**GLRaVs**	**GFKV**	**GFLV**
Argamussa	0	100	100	0
Batista Felanitx	0	100	20	0
Callet	0	100	22.22	0
Callet Negrella	0	71.42	42.85	0
Calop Blanc	0	100	100	0
Calop Negre	0	100	100	0
Calop Roig	0	100	100	0
Escursac	30.0	70	0	30
Esperó de Gall	0	100	100	88.8
Fernandella	37.5	5	25.0	0
Fogoneu	11.1	88.8	0	11.1
Gafarró	14.2	85.7	0	0
Galmeter	0	100	100	100
Giró Ros	60.0	40.0	0	0
Gorgollassa	10.0	40.0	0	90.0
Jaumes	12.5	87.5	25.0	75.0
Magdalena	0	100	0	0
Malvasia de Banyalbufar	31.2	68.7	6.2	6.2
Mamella Vaca	0	100	0	0
Mances de Capdell	0	94.4	50	44.4
Mancés de Tibus	70	10	0	20
Mandó	0	37.5	100	0
Manto negro	0	88.8	44.4	0
Mateu	0	100	0	0
Molinera	0	100	0	0
Moll	0	100	66.6	0
Quigat	0	100	100	0
Sabater	0	100	22.2	88.8
Sinso	0	100	22	0
Valent Blanc	0	100	58.3	100
Valent Negre	0	100	100	77.7
Vinater Blanc	0	100	100	0
Vinater Tinto	0	76.4	58.8	52.9

Supplementary material S2:

Nominal logistic model used to analyze the differences on the sanitary status among cultivars with the identity of the virus presented as response variable using JMP 10 software package.

Nominal Logistic Fit for infeccion

Whole Model Test

Model	-LogLikelihood	DF	ChiSquare	Prob>ChiSq
Difference	359,36461	384	718,7292	<,0001
Full	221,68686			
Reduced	581,05148			

RSquare (U)	0,6185
Observations (or Sum Wgts)	315

Converged by Objective

Effect Likelihood Ratio Tests

Source	Nparm	DF	L-R ChiSquare	Prob>ChiSq
Variedad	384	384	718,729224	<,0001

Generalized linear models (GLM) with binomial distribution with sanitation treatments as fix factor and Gorgollassa plants infection resulted as respond variable fit using JMP 10 software package.

Generalized Linear Model Fit

Response: virus presence

Distribution: Binomial

Link: Logit

Observations (or Sum Wgts) = 103

Whole Model Test

Model	-LogLikelihood	L-R ChiSquare	DF	Prob>ChiSq
Difference	1,66689276	3,3338	1	0,0679
Full	44,4716987			
Reduced	46,1385915			

Goodness Of Fit Statistic	ChiSquare	DF	Prob>ChiSq
Pearson	103,0000	101	0,4260
Deviance	88,9434	101	0,7988

Effect Tests

Source	DF	L-R ChiSquare	Prob>ChiSq
treatment	1	3,3337855	0,0679
